# Effect of Starch and Paperboard Reinforcing Structures on Insulative Fiber Foam Composites

**DOI:** 10.3390/polym16070911

**Published:** 2024-03-26

**Authors:** Gregory M. Glenn, Gustavo H. D. Tonoli, Luiz E. Silva, Artur P. Klamczynski, Delilah Wood, Bor-Sen Chiou, Charles Lee, William Hart-Cooper, Zach McCaffrey, William Orts

**Affiliations:** 1 United States Department of Agriculture, Agricultural Research Service, Western Regional Research Center, Bioproducts Research Unit, 800 Buchanan Street, Albany, CA 94710, USA; artur.klamczynski@usda.gov (A.P.K.); de.wood@usda.gov (D.W.); bor-sen.chiou@usda.gov (B.-S.C.); charles.lee@usda.gov (C.L.); william.hart-cooper@usda.gov (W.H.-C.); zach.mccaffrey@usda.gov (Z.M.); bill.orts@usda.gov (W.O.); 2 Forest Science Department, Federal University of Lavras, Lavras 37203-202, MG, Brazil; gustavotonoli@ufla.br (G.H.D.T.); lesilvaflorestal@gmail.com (L.E.S.)

**Keywords:** packaging foam, plastic foam, renewable, compostable, sustainable, starch, plant-based composites

## Abstract

Single-use plastic foams are used extensively as interior packaging to insulate and protect items during shipment but have come under increasing scrutiny due to the volume sent to landfills and their negative impact on the environment. Insulative compression molded cellulose fiber foams could be a viable alternative, but they do not have the mechanical strength of plastic foams. To address this issue, a novel approach was used that combined the insulative properties of cellulose fiber foams, a binder (starch), and three different reinforcing paperboard elements (angular, cylindrical, and grid) to make low-density foam composites with excellent mechanical strength. Compression molded foams and composites had a consistent thickness and a smooth, flat finish. Respirometry tests showed the fiber foams mineralized in the range of 37 to 49% over a 46 d testing period. All of the samples had relatively low density (*D_d_*) and thermal conductivity (*TC*). The *D_d_* of samples ranged from 33.1 to 64.9 kg/m^3^, and *TC* ranged from 0.039 to 0.049 W/mk. The addition of starch to the fiber foam (FF+S) and composites not only increased *D_d_*, drying time (*T_d_*), and *TC* by an average of 18%, 55%, and 5.5%, respectively, but also dramatically increased the mechanical strength. The FF+S foam and paperboard composites had 240% and 350% higher average flexural strength (*σ_fM_*) and modulus (*E_f_*), respectively, than the FF-S composites. The FF-S grid composite and all the FF+S foam and composite samples had equal or higher *σ_fM_* than EPS foam. Additionally, FF+S foam and paperboard composites had 187% and 354% higher average compression strength (*CS*) and modulus (*E_c_*), respectively, than the FF-S foam and composites. All the paperboard composites for both FF+S and FF-S samples had comparable or higher *CS,* but only the FF+S cylinder and grid samples had greater toughness (Ω_c_) than EPS foam. Fiber foams and foam composites are compatible with existing paper recycling streams and show promise as a biodegradable, insulative alternative to EPS foam internal packaging.

## 1. Introduction

The packaging and distribution of goods is a multi-billion dollar business worldwide, with nearly 500 million packages being transported every day using a myriad of different package configurations [1,2]. In 2022, 161 billion packages were shipped worldwide. That number is expected to reach 256 billion by 2027 [3]. Although there is no meaningful biodegradation of commodity plastics, which are mostly derived from non-renewable resources, they continue to play a major role in the packaging sector. More than 40% of the worldwide production of plastics is used for packaging, much of which is single-use [4,5]. A small percentage of plastics is reused/recycled, but roughly 80% is either landfilled, incinerated, or leaked into the environment [4,6].

For many commercial products such as small appliances, printers, etc., corrugated paperboard is used as an exterior packaging material, while plastic foam primarily from expanded polystyrene (EPS) or polypropylene (EPP) is used as interior packaging/cushioning material [4,7]. EPS foam is one of the preferred internal packaging foams because of its light weight, impact and moisture resistance, low cost, and ability to protect products from temperature extremes [7,8,9,10].

Despite its many advantages for internal packaging, EPS foam has become widely recognized for its negative impact on the environment [9,11]. EPS is very resistant to biodegradation [12]. While there are claims that 19–25% of EPS foam is recycled [13], that amount is disputed, in part because there are too few recycling centers available that process it [9]. Furthermore, EPS recycling is expensive partly due to its bulk and resistance to compaction [13,14,15]. These and other concerns have led several U.S. states and countries to enact legislation to ban single-use EPS foam products in an effort to phase out its use [16,17,18,19,20,21].

Alternatives to plastic foam packaging are being considered, using bioplastics such as poly(lactic acid) (PLA) and polyhydroxyalkanoate (PHA) as “drop-in” replacements for commodity plastics [11,22]. However, PHAs are still too expensive for single-use packaging, and PLA, like commodity plastics, degrades very slowly in marine and landscape environments [22]. PLA will biodegrade under humid conditions at elevated temperatures, but only a limited number of industrial composting operations are designed to handle PLA products [23]. Furthermore, while PLA can be foamed, the preferred foaming process is expensive due to the complexity and challenges involved in using supercritical fluids [24].

The most successful recycled/reused packaging material is paper and paperboard including corrugated paperboard used for external packaging. The EPA reported that paper and paperboard made up nearly 67% of the recycled municipal solid waste (MSW) materials in the U.S., while paper recycling in Europe exceeds 70% [25,26]. These cellulose-based products are biodegradable, derived from renewable resources, easy to reuse/recycle, and, unlike commodity plastics, will disintegrate and decompose if leaked into waterways or landscape environments [26]. There is growing interest in exploring and expanding the use of cellulose-based materials for internal packaging applications that can supplant EPS foam and be recycled or composted along with paperboard using well-established processing streams.

Interconnecting grid and honeycomb paperboard panels are examples of cellulose-based products designed primarily for internal packaging. These paperboard structures comprise a core consisting of empty square or hexagonal cells constructed in a grid or honeycomb pattern, respectively. The paperboard core may be sandwiched between two face sheets that adhere to the top and bottom surfaces and securely bind/anchor the core [27]. Grid/honeycomb paperboard is lightweight, has excellent compressive strength and shock and vibration resistance and is used extensively as cushioning material for transporting electronic equipment, appliances, furniture, etc., but lacks the insulation properties of EPS foam [27,28,29]. Abd Kadir et al. (2016) [30] filled the void spaces in the core of a paperboard honeycomb with low-density polyurethane foam to insulate and strengthen the walls of the core. The composite had superior compressive strength, but, like EPS foam, polyurethane foam is not compostable, and such composites would be difficult to recycle.

A foam that is compostable and recyclable can be made from aqueous cellulose fiber suspensions using a foaming agent [31,32]. Recently, a compostable foam made from cellulose fiber with excellent insulative properties was described that could be compression molded into distinct shapes or large panels needed for internal packaging [33]. However, by themselves, these fiber foams do not have the compressive strength or toughness that may be needed for many internal packaging applications [33,34]. Starch has been used to make biopolymer blends with excellent strength and toughness [35]. To our knowledge, there have been no studies reporting the properties of composites made from cellulose fiber foam and paperboard grids or other reinforcing structures that could provide the mechanical strength, toughness, and insulative properties EPS foam packaging provides. The objective of this study was to investigate the physical and mechanical properties of fiber foam/paperboard composites with and without a starch binder and explore their potential as an alternative to EPS foam packaging.

## 2. Materials and Methods

### 2.1. Materials

Pulped softwood fiber sheets were obtained from International Paper (Global Cellulose Fibers, Memphis, TN, USA) and produced at their Columbus, MS mill. The fiber was a Southern bleached softwood Kraft with a fiber length ranging from 3.8 to 4.4 mm and an ash content of 0.12%. Reagent-grade sodium dodecyl sulfate (SDS, Cas 151-21-3) was purchased from Thermo Fisher Scientific (Waltham, MA, USA). Paperboard (brown kraft cardboard chipboard (22 point with a thickness of 0.56 mm) was purchased from Magicwater Via GSD (Fontana, CA, USA). Brown kraft paperboard tubes (40 mm diameter × 100 mm length × 0.45 mm thickness) were purchased locally. Polyvinyl alcohol (PVA, Selvol 540, 88% hydrolyzed, 12% acetate, MW = 120,000) was purchased from Sekisui Chemical (Pasadena, TX, USA). Water-soluble pregelatinized waxy corn starch powder (Clearjel, Ingredion, Westchester, IL, USA) containing 0.2% ash, 0.1% protein, and <0.1% fat was obtained from Ingredion (Westchester, IL, USA). Expanded polystyrene (EPS) foam sheets (122 cm × 30.5 cm × 2.62 cm) were purchased locally.

### 2.2. Paperboard Support Elements

Three different support elements were prepared, consisting of an angular, cylindrical, and interlocking grid design. The angular elements were made by folding paperboard strips (26 mm in width and 52 mm in length) in half to form a 90-degree angle. Cylindrical elements were made by cutting paperboard tubes (40 mm dia.) to a length of 26 mm. The paperboard grid was made by assembling strips 26 mm in width with slots cut every 38 mm along the length into a grid pattern. The paperboard elements were embedded in the fiber foam, as described below.

### 2.3. Solution Preparation

An aqueous polyvinyl alcohol (PVA) solution (5%, *w*/*w*) was made by gradually adding PVA powder to cold water while continuously stirring and then slowly heating (95 °C) until the PVA was solubilized. Water was added to compensate for weight loss due to evaporation. A 29% (*w*/*w*) aqueous solution of SDS was made by combining SDS powder and water at room temperature and continuously stirring to achieve dissolution.

### 2.4. Foam Procedure

A low-moisture fiber foam formulation without starch (FF-S) was developed based on prior research (Table 1) [33]. The pulped fiber was prepared by first weighing the appropriate amount of pulp fiber (Table 1) and placing it in a blender containing approximately 2 L of warm (60 °C) tap water. The fiber was blended for approximately 30 s to disperse and hydrate the fiber. The fiber was allowed to hydrate for approximately 15 min before blending again for 30 s. The fiber mixture was then poured onto a screen (50 mesh) to allow drainage. The fiber was collected from the screen and compressed to expel excess water until the approximate combined weight of the fiber and water was reached for each sample formulation (Table 1). The final combined weight of water and fiber was adjusted by adding water to bring the mixture to the desired final weight.

The combined fiber and water sample was added to a 4 L mixing bowl of a planetary mixer (Model KSM 90, KitchenAid, Inc., St. Joseph, MI, USA). For the control sample, additional ingredients were added, as shown in Table 1. The initial weight of the mixing bowl and ingredients was recorded. Water was added occasionally during the mixing step to compensate for weight loss due to evaporation. Mixing started slowly (speed 3) and gradually increased to a speed of 10. A spatula was used to occasionally wipe down the bowl during mixing. The PVA and SDS both facilitated the dispersion of the fiber and prevented aggregation. Once a foam was produced, mixing was paused to measure the wet density (*D_w_*) of the foam and to add water to compensate for any weight loss that occurred due to evaporation. *D_w_* was determined by filling a cup to level with wet foam and recording the weight and volume. The foam was mixed until the desired *D_w_* (Table 2) was achieved.

The mixing procedure for the fiber foam with starch (FF+S) sample was similar to FF-S except for the fact that the water-soluble starch powder was gradually added to the mixing bowl only after the ingredients had started to foam. The starch powder was slowly added to the foam while mixing to ensure that the starch was properly dispersed and solubilized in the foam mixture. Starch tended to reduce the foam volume so higher amounts of water and foaming agent were added to compensate for the reduction in foam volume (Table 1). Notwithstanding the additional amount of water and foaming agent, the final *D_w_* of the foam containing starch was higher than foam without starch (Table 2).

The air uptake volume (*Va*) of the foam was calculated using Equation (1), where *V_system_* is the volume of the ingredients before foaming, and *V_air_* is the bulk volume of the foamed material. The *V_system_* was derived from the specific gravity of each component. Specific gravity values were obtained using a helium gas displacement pycnometer (Micromeritics, model AcuPyc II 1340, Norcross, GA, USA). The specific gravity values (g/cm^3^) of the dry ingredients used in calculations included the following: fiber (1.61); PVA (1.30); SDS (1.01); and starch (1.46). The specific gravity of water (1.0 g/cm^3^) was used to determine the volume of water added, including in the SDS and PVA solutions. The *V_system_* values (cm^3^) for the control and starch formulations, as shown in Table 1, were 234 cm^3^ and 288 cm^3^, respectively. The *V_air_* values for the control and starch formulations were 2032 cm^3^ and 1731 cm^3^, respectively.

Va (%) = V_air_/V_system_ × 100 (1)

### 2.5. Compression Molding

**Figure 1:**
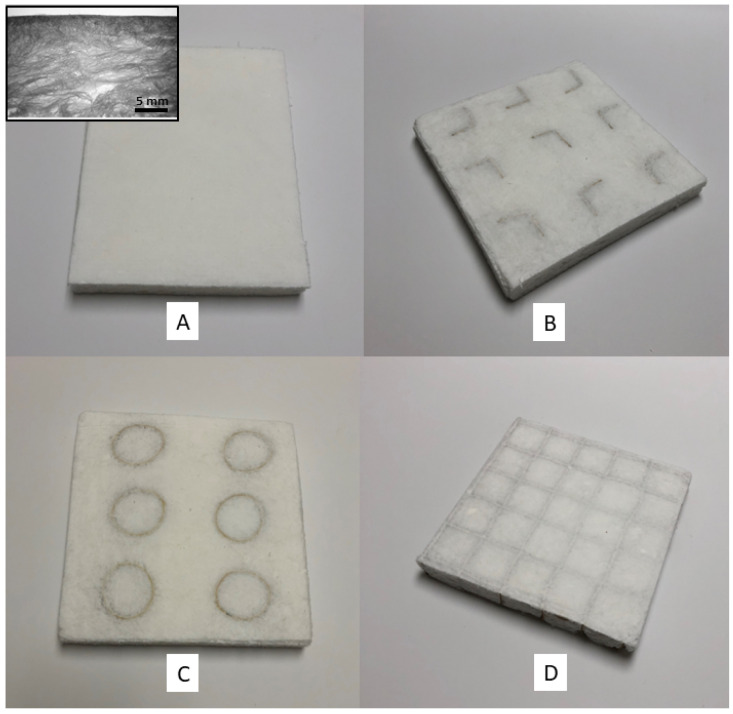
Photographs of fiber foam and paperboard composite samples where the platen assemblies were removed only after the drying process. Samples included the fiber foam (**A**) and composites containing angled (**B**), cylindrical (**C**), and grid (**D**) paperboard elements. Insert in A is a micrograph of cross-sectional view of foam. Scale bar = 5 mm.

The mold assembly consisted of upper and lower porous platen assemblies, as described previously [33]. The volume of the mold cavity was calculated from dimensional measurements. The weight of foam required to overfill the mold to 135% of the mold volume was calculated from the *D_w_* values. After loading the mold with excess foam, the upper platen was lowered, which compressed the foam, causing it to flow and conform to the mold and form a skin on the upper and lower surfaces that were in contact with the platens. For the fiber foam/paperboard composites, approximately 80% of the foam was added to the mold. A spatula was used to spread the foam uniformly inside the mold. The paperboard elements were then carefully pressed into the foam in a prescribed pattern (Figure 1). The remaining quantity of foam was spread on top of the paperboard elements, and the upper platen was lowered, which compressed the foam, causing it to flow and fill any voids as previously described [33]. The intact platen assembly was placed in an oven for drying.

### 2.6. Drying

The foam samples were oven-dried at 80 °C. The weight loss was monitored by periodically weighing the samples. The end time of drying was recorded as the point where less than 0.15% of the initial weight of the foam was lost over a 30 min drying interval. The initial and end times for drying were used to record the total drying time. Once drying was completed, the platen assemblies were dismantled, and the molded foam sample was removed and stored in a plastic bag at room temperature until further testing.

### 2.7. Mechanical Properties

The compressive and flexural properties of the samples were measured using a universal testing machine (Model ESM303, Mark-10, Copiague, NY, USA). The compressive properties of dry foams were measured on samples cut to dimensions approximately (5 cm × 5 cm) as per ASTM standard D-1621 [36]. Final dimensions were measured using calipers. The samples were conditioned for 48 h in a chamber with a small circulating fan. The relative humidity of the chamber was maintained near 50% using a saturated salt solution (Mg(NO_3_)·6H_2_O) as previously described [37]. Compression tests were performed using a deformation rate of 12.5 mm/min as per established methods (ASTM D 1621) [36]. Compressive strength was recorded as the stress at the yield point before 10% strain. The fiber foam without starch did not have a clear yield point, so the stress at 10% strain was recorded as the *CS* as per ASTM standard [36]. Samples were subjected to five load/unload cycles up to 50% strain using a deformation rate of 2.5 mm/min. The area under the loading curve was used to calculate toughness (Ω). A minimum of five replicates were made for each treatment.

### 2.8. Flexural Tests

Three-point flexural tests were performed using samples cut to dimensions approximately (20 cm × 5.0 cm × 2.6 cm). The final width and thickness measurements of samples were recorded using calipers. The flexural tests were performed using a deformation rate of 2.5 mm min^−1^, a span distance of 152 mm, and a span/depth ratio of 5.85. Flexural stress (*σ_f_*) and strain (ε*_f_*) were calculated as per ASTM D790 [38].

### 2.9. Physical Properties

The dry bulk densities of the samples were determined from volume and weight measurements of oven-dried specimens [39]. Helium gas displacement pycnometry was used to determine the specific density (*dn*) of the foam solids. Porosity (*P*) was determined from the bulk density of the foams (*da*) and the specific density of the foam (*dn*) using Equation (2), which was obtained from the simple mixing rule with a negligible gas density [39]. The *dn* value of the foam solids from gas pyncnometry was 1.55 g/cm^3^.

*P(%) = 100 × (1 − da/dn)* (2)

### 2.10. Thermal Conductivity

Thermal conductivity was measured at a mean temperature of 22.7 °C on panel samples for each treatment according to standard methods (ASTM C-177-85) [40] using a thermal conductivity instrument (model GP-500, Sparrell Engineering, Damarascotta, ME, USA). Readings were taken at 1 h intervals as the instrument approached thermal equilibrium.

### 2.11. Respirometry

An automated respirometer system (Microoxymax, Columbus Instruments, Columbus, OH, USA) was used to monitor the mineralization of the fiber foams as per ASTM methods (D5338) with only minor modifications. Compost purchased locally was sieved (14 mesh) and stored overnight for moisture equilibration. Moisture content was determined gravimetrically by drying 10 g samples at 105 °C for 16 h. Fiber foam samples (with and without starch) were cut into small pieces (<5 mm) and weighed (~0.5 g) to the nearest 0.1 mg. The samples were added to a reaction jar along with compost (24.5 g), taking care to ensure uniform mixing. The moisture content was adjusted to 58% by adding water before beginning a run. Samples were kept for two days at 30 °C before raising the temperature to 58 °C. During the run, the CO_2_ concentration was measured at 2 h intervals. Water (2 mL) was added daily to maintain the moisture content range between 50 and 60%. The carbon content of the samples was determined using a CHN Analyzer (Elementar Vario el Cube, Ronkonkoma, NY, USA). The theoretical percent biodegradation was calculated as the ratio of the moles of carbon in the sample versus the accumulated moles of CO_2_ produced utilizing the ideal gas law as previously described [41]. 

### 2.12. Microscopy

Light micrographs were taken using a digital microscope (Dino-Lite model AM3113, Torrance, CA, USA) equipped with image capture software (Dinocapture 2.0). Cross-sectional slices (1 cm) of fiber foam samples were cut using a scroll saw. Backlighting was used to provide higher-contrast photomicrographs.

### 2.13. Thermogravimetric Analysis (TGA)

A Mettler Toledo TGA/DSC 3+ thermogravimetric analyzer (Greifensee, Switzerland) was used to determine the thermal stability of the foams. Each sample was first conditioned at 23 °C in a 50% relative humidity chamber for at least 48 h. The 8–11 mg sample was then heated from 30 °C to 650 °C in an alumina crucible at 10 °C/min. The sample chamber was purged with nitrogen gas at 40 cm^3^/min.

### 2.14. Statistical Analysis

The data were analyzed by a one-way analysis of variance. A Tukey–Kramer Post Hoc test (α < 0.05) was used to determine differences between treatment means. Significant differences were noted by the different letters following the mean values within rows in data tables.

## 3. Results and Discussion

Paperboard panels made with paperboard elements, including honeycomb, grid, and multi-cell lattice designs, can provide the mechanical strength and shock resistance needed in many internal packaging applications, but they lack the thermal insulation provided by plastic foams that may be needed for some packaging systems [27,29]. In the present study, cellulose fiber-based foam panels, both with and without a binder (starch), were produced that had good insulation properties and were attractive, flat (no warping, Figure 1), and had uniform thickness (Table 2). The foam comprised a core of entangled fibers with skin on the surface (see insert, Figure 1A). Three different paperboard reinforcing elements (angle, cylindrical, and interlocking grid) were embedded into the wet foam, which was then dried. The paperboard elements were tested to demonstrate that both interlocking and non-interlocking paperboard elements could be used with the foaming process (Figure 1). Non-interlocking elements, i.e., angle and cylindrical elements, were essentially anchored into position by the foam itself as it dried (Figure 1). This eliminated the need to glue face sheets onto the surfaces of the panels, as is common in paperboard honeycomb panels [29].

The foaming procedure provides the flexibility to test different permutations in the paperboard composite concept. For instance, different geometrical designs, fewer or more elements, varied spatial arrangement, and a variety of different paperboard thicknesses could be tested to help optimize the mechanical properties, density, and thermal conductivity of the fiber foam panels. The total weight of each set of angles, cylindrical, and grid elements used in making panels was 4.6, 6.2, and 22.4 g, respectively. Coincidentally, the *D_d_* and thermal conductivity (*TC*) of the foam composites were positively correlated with the weight of the paperboard element sets used (Table 2).

All the composites tested had relatively low thermal conductivity and density but there were still significant differences among the samples themselves (Table 2). The EPS foam had the lowest density (*D_d_*) and thermal conductivity (*TC*), while the grid composites had the highest. The volume (*Va*) of the wet foam and the pore volume of the dry foam (*%P*) were inversely related to the wet (*D_w_*) and dry (*D_d_*) densities (Table 2). The addition of starch to the foam formulation (FF+S) decreased the *Va* and *%P,* resulting in higher *D_w_* and *D_d_* than the FF-S sample. The decrease in *Va* and *%P* and the concomitant increase in *D_d_* resulted in greater flexural and compressive strength (Table 3 and Table 4).

The drying time (*T_d_*) required at 80 °C was considerable (Table 2), but other approaches to drying the foam could be explored. For instance, sample thickness could be reduced, or other efficient drying technologies could be tested, including ambient air drying and solar-assisted, infrared-assisted, microwave-assisted, and similar hybrid drying technologies [42]. 

**Figure 2:**
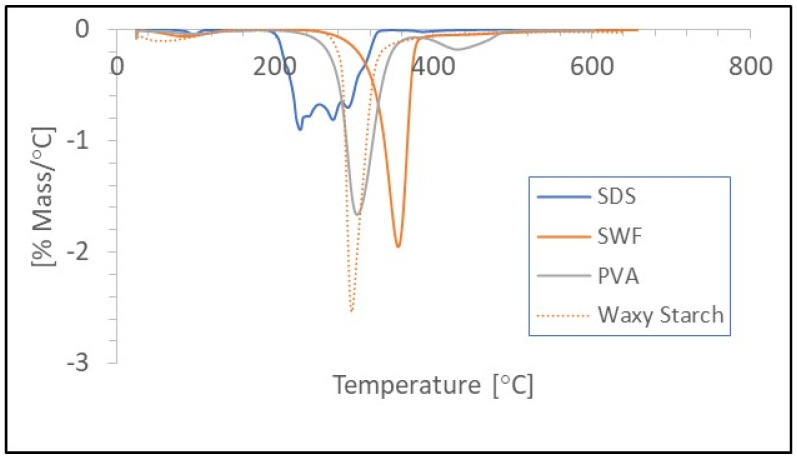
Graph of the DTG curves for Sodium dodecyl sulfate (SDS), polyvinyl alcohol (PVA), starch, and softwood fiber (SWF) from thermogravimetric analysis.

TGA analysis was performed to study the thermal decomposition properties of the individual foam components. These data were used to establish an upper temperature range for the drying oven. The derivative of the wt.% curve was used to determine the decomposition temperatures as shown in Figure 2. The TGA data indicate that the least thermally stable component was SDS which had a decomposition temperature of 237 °C. Decomposition temperatures for starch, PVA, and SWF were 301, 308, and 354 °C, respectively.

Based on the TGA results, oven drying temperatures in excess of 200 °C might be considered the upper temperature range for drying conditions. However, in preliminary drying tests, a strong odor was detected when samples were dried at only 120 °C. There was little or no odor detected when the samples were dried at 80 °C. The odor produced at 120 °C was attributed to the greater thermal instability of SDS in an aqueous environment. In the presence of water, SDS is reported to degrade into fatty alcohols and sodium sulfate after prolonged heating at relatively low temperatures [43]. Alternative foaming agents have been used for making cellulose foam and could be evaluated to determine whether they are more thermally stable and/or compatible with alternative drying methods [31].

The drying times (*T_d_*) for samples tested ranged from 333 to 553 min (Table 2). The addition of starch to the foam formulation (FF+S) significantly (paired *t*-test, t = 6.44 × 10^−4^) increased the drying time (*T_d_*) compared to samples without starch (FF-S, Table 2). The results highlight the effect a single ingredient can have on *T_d_* and underscore the importance of assessing the impact different ingredients or even the ratio of ingredients may have on processing parameters.

**Figure 3:**
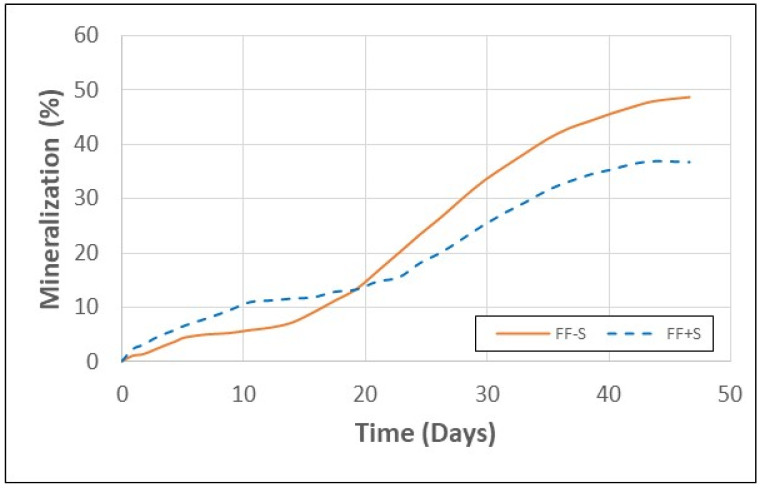
Mineralization rate of fiber foam samples both with and without starch.

As previously mentioned, legislative measures are being taken to phase out the use of EPS foam primarily in single-use packaging due to the resistance of EPS foam to biodegradation and its negative environmental impact [44]. In contrast to EPS foam, cellulose fiber-based products, such as paper and paperboard, are renewable, recyclable, compostable, and biodegradable [26]. Under favorable composting conditions, paper waste partially mineralizes to CO_2_ while the residue forms humus, which is an excellent soil amendment [45]. Humus can slowly degrade further by fungi, bacteria, and soil organisms such as earthworms. The rate at which paper fiber biodegrades is dependent upon various factors, including how the fiber was originally processed, the lignan content, the type of paper additives used, and the environmental conditions [45]. Respirometry data from the present study showed that FF-S and FF+S samples mineralized in the range of 37–49% over a 46-day period (Figure 3). This is consistent with previous studies that report a 43–79% rate of mineralization for different papers over a 45-day period under composting conditions [46]. 

Cellulose fiber-based packaging materials are desirable partly because they can be reused and recycled. Paper products are reported to be recyclable up to seven times [26]. There is a well-established infrastructure for recycling paper and paperboard, especially in developed countries [26]. Fiber foam/paperboard composites are well suited for recycling using existing paper recycling streams partly because all the components used in making the fiber foam composites are already used in varying amounts in paper products.

The fiber foam and composites pose much less of an environmental concern compared to EPS foam, in part because all the ingredients, including the cellulose fiber, starch, SDS, and PVA, are biodegradable, at least to various extents. The chemical interaction of the ingredients is likely to be carried out mostly via hydrogen bonds between the hydroxyl groups on the fibers, starch, and PVA. These bonds can be easily disrupted by SDS under moist conditions encountered in composting environments, which then facilitates microbial access and biodegradation. It is well established that cellulose fiber and starch readily biodegrade in many environments. The fate of SDS in the environment has also been investigated [47]. Among its many applications, SDS is used as a fat emulsifier and as an ingredient in cosmetics, pharmaceuticals, and toothpaste [48]. It is also used extensively in deinking recycled paper and in various other processes of paper production [49]. In aerobic and anaerobic environments, SDS readily biodegrades into simple, nontoxic components and does not persist in the environment [47].

The most persistent component of fiber foams is PVA. PVA is a biocompatible polymer and can be manufactured economically from non-petroleum routes [50]. The environmental fate of PVA has been a subject of wide debate [37,51,52]. PVA is commonly used in the paper and textile industries as a sizing agent and is known to biodegrade in the presence of specific microorganisms [50,52]. The PVA degrading microorganisms are present in wastewater and compost environments [51,52,53]. Although PVA can degrade at rates similar to cellulose under optimal conditions [50], its biodegradation is much slower in many environments. For instance, PVA is known to accumulate as a pollutant in wastewater [52]. The PVA used in fiber foam formulations could be removed if necessary to further reduce the environmental footprint of the fiber foam. PVA-free fiber foam has been reported, but it requires a higher amount of water, which may lengthen the drying time [33]. Regardless of whether they contain PVA or not, the paperboard/fiber foam composites provide a more sustainable and environmentally benign option compared to EPS foam.

**Figure 4:**
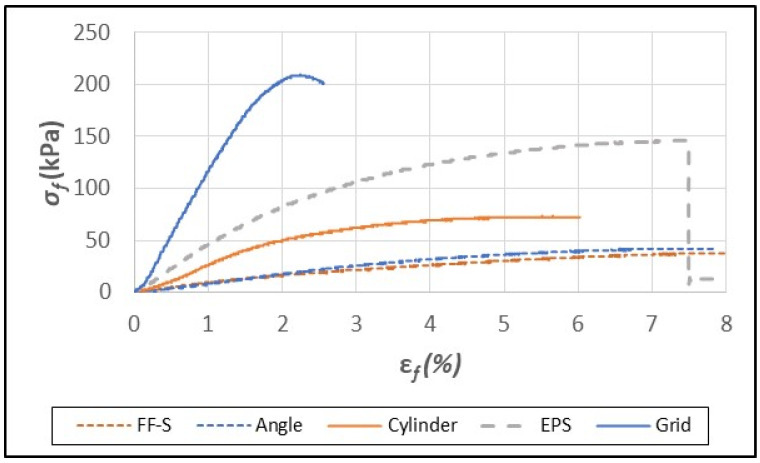
Typical flexural stress (σ_f_)/strain (ε_f_) curves for fiber foam without starch (FF-S), EPS foam, and FF-S composites containing paperboard elements (angle, cylinder, and grid).

**Figure 5:**
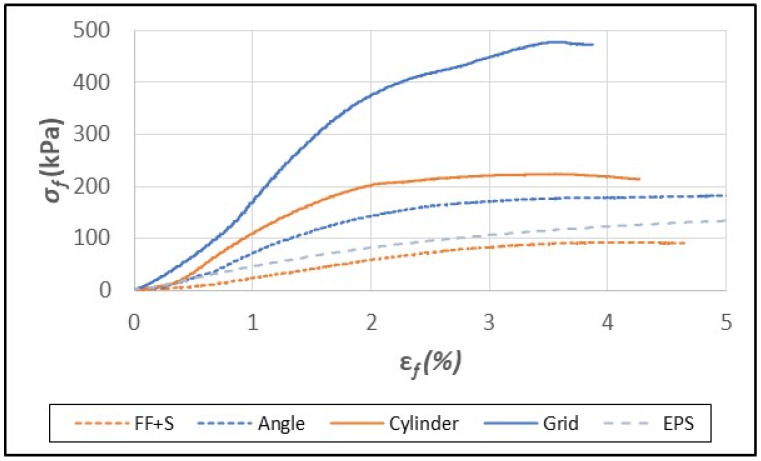
Typical flexural stress (σ_f_)/strain (ε_f_) curves for fiber foam with starch (FF+S), EPS foam, and FF+S composites containing paperboard elements (angle, cylinder, and grid).

As previously mentioned, paper/paperboard grid/honeycomb packaging has outstanding toughness and provides excellent shock resistance in internal packaging applications [29]. The fiber foam composites, which were shown earlier to have good insulative properties, were tested under flexural and compressive strain as a means of assessing their mechanical strength and suitability as a replacement for EPS foam. In flexural tests, EPS foam samples failed abruptly in the range of 7–8% strain (ε*_fM_*, Table 3, Figure 4). The fiber foam samples and composites, however, typically reached a peak force of resistance, and then yielded and eventually formed a bend but did not break except for the grid composite (Figure 4 and Figure 5).

The interlocking grid structure provided reinforcement against flexural strain, resulting in much higher strength (*σ_fM_*) and modulus (*E_f_*) values compared to the other composites (Table 3). The breakage of the grid structure sometimes occurred due to tearing that originated from the slots that were cut to form the interlocking grid. The FF-S foam and composites had lower flexural strength (*σ_fM_*) than EPS foam except for the FF-S grid sample (Table 3, Figure 4).

In contrast to the FF-S samples, the FF+S foam composites all had flexural strength (*σ_fM_*) and modulus (*E_f_*) values in the same range or higher than the EPS foam (Table 3, Figure 5). The greater strength in samples containing starch is likely due to the ability of starch to act as an adhesive that binds fibers together and to the paperboard elements thus helping to reinforce the foam component and anchor the paperboard elements in place. The tearing and failure of the grid structure observed in the FF-S sample was less apparent in the FF+S grid sample (Figure 5). This may have been due to the higher interfacial forces exerted between cellulose fibers and the polymer matrix as well as the binding effect of starch that helped to strengthen the walls of the paperboard elements and better distribute the flexural stress throughout the structure [54].

**Figure 6:**
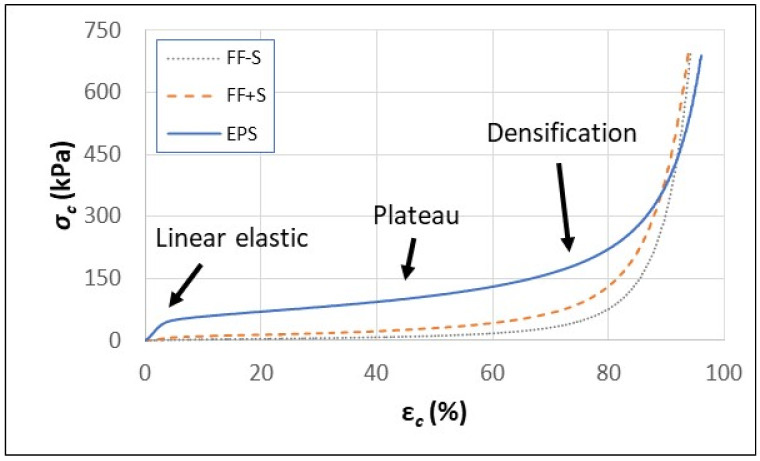
Stress–strain compression curves for EPS foam, fiber foam without starch (FF-S), and fiber foam with starch (FF+S).

The behavior of EPS foam under compressive strain (ε*_c_*) was very different than its behavior under flexural strain (compare Figure 4 and Figure 6). Compression stress/strain curves over an extremely large range (0–95%) in ε*_c_* were obtained for EPS foam, FF-S, and FF+S fiber foam samples (Figure 6). The compression curves for the FF-S and FF+S samples revealed a compression behavior similar to the EPS foam and other elastomeric foams (Figure 6) [55,56]. A linear elastic region was observed at the beginning of the curves, followed by a plateau region where *σ_c_* values increased at a relatively slow rate compared to the change in *ε_c_*. Under very high *ε_c_* values, the foams continued to densify and behave more like a solid than a foam (Figure 6). Despite densification, the foams remained intact and did not shatter or fracture. The results indicate that even under extreme levels of compaction/densification, the fiber foams behave as elastomeric foams similar to EPS foams and are able to withstand excessive *ε_c_* without fracturing. The compression results also showed that foam formulations containing starch (FF+S) had higher *σ_c_* values than FF-S, although neither of the fiber foam samples were in the range of the EPS foam sample (Figure 6).

Embedding paperboard elements (angle, cylinder, and grid) in the fiber foam (FF-S, FF+S) as a reinforcement had a significant effect on their compressive properties (Table 4). Compressive strength (CS) and toughness (*Ω_c_*) have been used to assess the ability of a material to resist compressive strain (*ε_c_*) and absorb shock during shipping [29,31]. The paperboard elements increased the *CS* and *Ω_c_* values, which were measured at 10% and 50% *ε_c_.* (Table 4). The angle paperboard composites had compressive strength (*CS*) and modulus (*E_c_*) values in the range of EPS foam but had lower *Ω_c_* values (Table 4). Meanwhile, the composites containing cylinder and grid elements had significantly higher *CS, E_c,_* and *Ω_c_* values than EPS foam, whether they contained starch or not (Table 4). The FF+S composites had higher mean values for *CS*, *E_c,_* and *Ω_c_* than the corresponding FF-S samples (Table 4).

**Figure 7:**
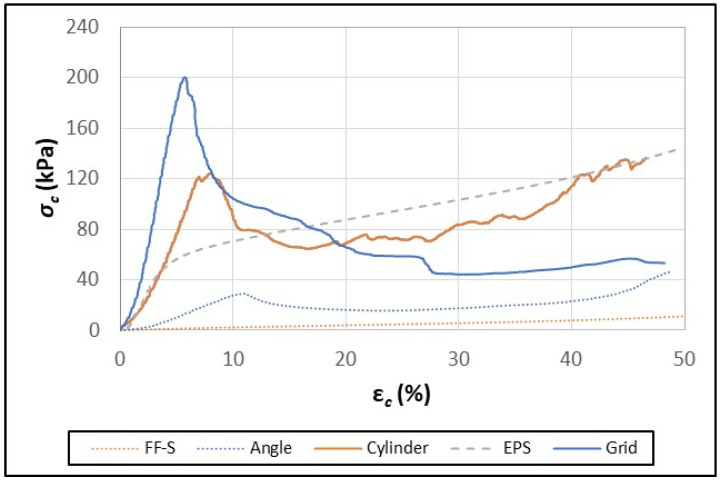
Typical compressive stress–strain curves for EPS foam, fiber foam without starch (FF-S), and FF-S composites containing angular, cylindrical, or grid paperboard elements.

**Figure 8:**
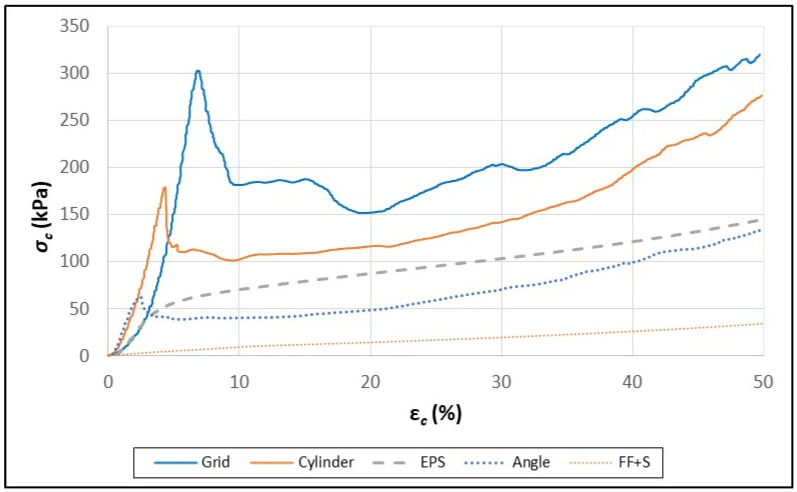
Stress–strain curves for starch containing fiber foam (1), starch/fiber/paperboard composites (angle = 2, cylinder = 3, grid = 4), and EPS foam (dashed line). These data are for the first (initial) stress/strain cycle.

Although the FF-S and FF+S samples behaved like typical elastomeric foams under a wide range of compressive strain (Figure 6), the paperboard composites did not (Figure 7 and Figure 8). Li et al. (2022) [29] reported that, initially, the compression curves for paperboard honeycomb panels increased linearly, peaked, and then decreased linearly before plateauing. A similar pattern was observed in the data for both the FF-S and FF+S paperboard composites (Figure 7 and Figure 8). The rapid drop in the linear region of the compression curves was due to the failure/buckling of the walls of the paperboard elements under excessive compressive strain (ε_c_) [29].

The differences observed between the FF-S and FF+S samples were greater than might be expected from the sum of the fiber foam and the individual paperboard elements themselves. This was particularly evident for the FF-S and FF+S cylinder and grid samples, which had a difference of 66 and 108 kPa, respectively (Table 4). 

It was somewhat surprising that the *σ_c_* values for the FF-S grid sample decreased continuously until almost 30% ε*_c_* before plateauing (Figure 7). Observation of the grid sample during compression tests revealed that the FF-S grid structure was not well anchored compared to the FF+S sample. As such, the walls of the FF-S grid were more easily able to dislodge and bend slightly at an angle thus preventing proper uniaxial loading. Furthermore, the slots cut for assembling and interconnecting the walls that formed the grid provided sites for tearing to occur as excessive compressive strain was applied.

In the FF+S compression curves, the *σ_c_* values did not plateau horizontally as reported previously for paperboard structures [29] but rather continued to rise as ε_c_ increased (Figure 8). The higher *σ_c_* values for the FF+S composites were likely due to the binding properties of starch that helped anchor the paperboard elements and allow better uniaxial loading. Additionally, when the paperboard elements were initially embedded in the wet foam, they became saturated and wet. The starch contained in the liquid phase likely made the paperboard elements stiffer and stronger upon drying.

## 4. Conclusions

Fiber foam and fiber foam/paperboard composites provide the thermal and mechanical properties needed in many packaging applications and that is currently provided by the EPS foam packaging slated to be phased out by legislative mandate. The composites do not require the use of face sheets as with honeycomb paperboard panels. The fiber foam process allows for the incorporation of starch as a binder and the use of both non-interlocking and interlocking reinforcing paperboard elements with different designs, spatial arrangement, and weight. Specific mechanical properties, densities, and thermal conductivity in the range of EPS foam can be targeted by varying the number and placement of paperboard elements and/or by incorporating a starch binder. Of the paperboard elements tested, composites made with cylindrical paperboard elements had the best overall performance in terms of flexural/compressive strength, modulus, and toughness while still maintaining a low density and thermal conductivity. The fiber foam/paperboard composites contained ingredients that are already used in the paper industry. This makes the foam composites more suitable for recycling in existing paper recycling streams. Further research is needed to identify a more thermally stable foaming agent and a more efficient drying technology that could reduce the time and energy needed for drying.

## Figures and Tables

**Table 1:** Formulations of fiber foam (FF) samples with and without starch (+S and −S, respectively). The percentage of each ingredient is included in the parentheses.

Sample	FF (−S)	FF (+S)
Fiber	50 g	50 g
	(19.7%)	(15.9%)
Water	100 g	150 g
	(39.4%)	(47.6%)
PVA (5% soln)	100 g	100 g
	(39.4%)	(31.7%)
SDS (29% soln)	4 g	8 g
	(1.57%)	(2.54%)
Starch	0 g	7 g
		(2.22%)

**Table 2:** Wet density (D_w_), foam volume (Va), drying time (T_d_), thickness (T), dry density (D_d_), porosity (P), and thermal conductivity (TC) of wet and dry fiber foam with and without starch (FF+S and FF-S, respectively) and composites containing paperboard elements (angle, cylinder, and grid) prepared using a planetary mixer. EPS = expanded polystyrene.

Sample	FF-S	FF+S	FF-S Angle	FF+S Angle	FF-S Cyl.	FF+S Cyl.	FF-S Grid	FF+S Grid	EPS Foam
*D_w_* (kg/m^3^)	125 ^a^*	182 ^b^	N/A	N/A	N/A	N/A	N/A	N/A	N/A
*Va* (%)	869 ^a^	601 ^b^	N/A	N/A	N/A	N/A	N/A	N/A	N/A
*T_d_* (min)	336 ^a^	528 ^c^	333 ^a^	510 ^c^	311 ^a^	529 ^c^	399 ^b^	553 ^c^	N/A
T (cm)	2.66 ^a^	2.62 ^a^	2.63 ^a^	2.63 ^a^	2.62 ^a^	2.61 ^a^	2.70 ^a^	2.65 ^a^	2.61 ^a^
*D_d_* (kg/m^3^)	33.1 ^b^	39.1 ^c^	35.9 ^b,c^	44.9 ^d^	39.1 ^c^	44.9 ^d^	57.1 ^e^	64.9 ^f^	14.1 ^a^
*P* (%)	97.9 ^a^	97.5 ^a^	N/A	N/A	N/A	N/A	N/A	N/A	98.6 ^b^
*TC* (W/mK)	0.039 ^a,b^	0.043 ^a,b,c^	0.042 ^a,b^	0.044 ^b,c,d^	0.042 ^a,b^	0.044 ^b,c,d^	0.048 ^c,d^	0.049 ^d^	0.038 ^a^

* Mean values within rows followed by a different letter are significantly different (*p* < 0.05).

**Table 3:** Flexural strength (σ_fM_), strain (ε_fM_), and modulus (E_f_) of fiber foam (FF) with and without starch (+S and −S, respectively), fiber foam composites containing paperboard elements (angle, cylinder, grid), and EPS foam.

Sample	FF-S	FF+S	FF-S Angle	FF+S Angle	FF-S Cyl.	FF+S Cyl.	FF-S Grid	FF+S Grid	EPS Foam
*σ_fM_* (kPa)	30.8 a*	94 bc	36 a	173 d	67 ab	224 e	191 de	460 f	133 cd
ε*_fM_* *(%)*	5.0	3.57 ab	5.0	4.18 ab	5.0	4.40 ab	2.90 a	3.29 a	5.00 c
*E_f_ (MPa)*	1.12 a	4.32 ab	1.21 a	9.45 bc	2.57 a	11.9 c	10.6 c	22.9 d	4.29 a

* Mean values (*n* = 5) within rows followed by a different letter are significantly different (*p* < 0.05).

**Table 4:** Compressive strength (CS) strain (ε_c_) and modulus (E_c_) of fiber foam (FF) with and without starch (+S and −S, respectively) and fiber foam composites containing paperboard elements (angle, cylinder, and grid).

Sample	FF-S	FF+S	FF-S Angle	FF+S Angle	FF-S Cyl.	FF+S Cyl.	FF-S Grid	FF+S Grid	EPS Foam
*CS* (kPa)	1.6 ^a^*	10 ^ab^	30 ^abc^	63 ^c^	121 ^d^	187 ^e^	192 ^e^	305 ^f^	55 ^bc^
ε_c_ (%)	10 ^e^	9.22 ^e^	6.0 ^cd^	2.1 ^a^	6.8 ^d^	3.9 ^abc^	4.5 ^bc^	3.7 ^ab^	4.0 ^cde^
*E_c_* (MPa)	0.016 ^a^	0.16 ^a^	0.70 ^a^	3.4 ^bc^	2.1 ^ab^	5.0 ^bc^	5.1 ^bc^	8.7 ^d^	1.7 ^ab^
*Ω_c_* (J) ε_c_ = 10%	0.0056 ^a^	0.041 ^a^	0.13 ^ab^	0.24 ^b^	0.46 ^c^	0.74 ^d^	0.77 ^d^	1.1 ^e^	0.34 ^c^
*Ω_c_* (J) ε_c_ = 50%	0.14 ^a^	0.63 ^a^	0.66 ^a^	2.1 ^b^	2.2 ^b^	4.8 ^d^	2.5 ^bc^	6.2 ^e^	3.1 ^c^

* Mean values (*n* = 5) within rows followed by a different letter are significantly different (α < 0.05).

## Data Availability

Data are contained within the article.
